# Design of a “Clean-Label” Gluten-Free Bread to Meet Consumers Demand

**DOI:** 10.3390/foods10020462

**Published:** 2021-02-20

**Authors:** Marco Montemurro, Erica Pontonio, Carlo Giuseppe Rizzello

**Affiliations:** 1 Department of Soil, Plant, and Food Science, University of Bari Aldo Moro, 70126 Bari, Italy; marco.montemurro@uniba.it (M.M.); erica.pontonio@uniba.it (E.P.); 2 Department of Environmental Biology, Sapienza University of Rome, 00185 Rome, Italy

**Keywords:** gluten-free bread, sourdough, clean-label

## Abstract

The market of gluten-free (GF) products has been steadily increasing in last few years. Due to the technological importance of gluten, the GF food production is still a challenge for the industry. Indeed, large quantities of fat, sugars, structuring agents, and flavor enhancers are added to GF formulations to make textural and sensorial characteristics comparable to conventional products, leading to nutritional and caloric intake imbalances. The formulation of the novel “clean-label” GF bread included a commonly used mixture of maize and rice flour (ratio 1:1) fortified with selected protein-rich flours. Naturally hydrocolloids-containing flours (psyllium, flaxseed, chia) were included in the bread formulation as structuring agents. A type-II sourdough was obtained by using a selected *Weissella cibaria* P9 and a GF sucrose-containing flour as substrate for fermentation to promote the exo-polysaccharides synthesis by the starter lactic acid bacterium. A two-step protocol for bread-making was set-up: first, the GF sourdough was fermented (24 h at 30 °C); then, it was mixed with the other ingredients (30% of the final dough) and leavened with baker’s yeast before baking. Overall, the novel GF bread was characterized by good textural properties, high protein content (8.9% of dry matter) and in vitro protein digestibility (76.9%), low sugar (1.0% of dry matter) and fat (3.1% of dry matter) content, and an in vitro predicted glycemic index of 85.

## 1. Introduction

Although the consumption of bread has showed a progressive decrease in recent decades, bread is considered one of the most important staple foods in the world. Its consumption in the last years was reported to be approximately 60 kg per capita per year in the Western countries [1].

Bread is commonly made using wheat flour, in which gluten is the main protein source. However, a growing part of the population is affected by several diseases related to gluten intake. Indeed, celiac disease (CD) is the most common and increasing food intolerance, affecting approximately 1% of the worldwide population [2]. In addition to CD, there are several disorders related to gluten intake, called gluten-related disorders, which include non-celiac gluten sensitivity (NCGS), dermatitis herpetiformis, wheat allergy, gluten ataxia, and other chronic inflammatory diseases (e.g., inflammatory bowel diseases, IBD), usually co-occurring with CD [3,4].

The primary treatment to avoid complications related to gluten assumption is the strict adherence to a gluten-free diet (GFD) [5]. Overall, among GF ingredients, corn and rice flours are extensively used to make GF bread [6]. However, several technological issues lead to difficulties in the production of GF bread. Indeed, gluten is the main structuring agent in wheat dough and confers its baking quality [7]. Several approaches were investigated to overcome the absence of wheat protein network, including the use of a wide range of ingredients and additives [8,9]. Structuring ingredients are usually classified in three different categories, which include (i) water-binding and film-forming ingredients (e.g., hydrocolloids or thickening agents), (ii) structure-forming, volume-filling, and taste-giving ingredients (e.g., proteins, fats, and low molecular weight carbohydrates), and (iii) surface-active substances (e.g., emulsifiers). However, the inclusion of many ingredients and additives give the perception of a high processed food, thus leading to consumers’ dissatisfaction. Indeed, in recent years, the consumer’s choice is continuously evolving toward a niche of products considered safer, which are referred to as “clean-label products” [10,11]. “Clean-label” is not a scientific definition, but it is a popular definition broadly accepted by the food industry, consumers, the scientific community, and even regulatory agencies. The definition refers to products made by using as few ingredients as possible, making sure they could be easily identified by the consumers as no-artificial, no-synthetic, wholesome ingredients [10,11].

The inclusion of non-wheat plant ingredients and the application of new technologies were recently proposed to overcome this issue. Being water-binding, film-forming, and a source of protein, fiber, and minerals [12,13], the use of pseudo-cereals and pulses in breadmaking has largely been encouraged, both in gluten-containing and gluten-free products [14,15,16].

In addition to flours deriving from legumes and pseudo-cereals, whose importance in the GF sector is mainly related to the nutritional and functional properties, other flours gained the attention of the producers as texture improvers. Psyllium flour is reported as one of the most important ingredients already used in commercial GF bread due to its structuring properties [9,17]. Other seeds (or derived flours) such as chia and flaxseed have been proposed thanks to their water-binding capacity and production of mucilage [18], which favor the workability and cohesiveness of the GF doughs, and the hydration and softness of the GF breads.

In addition to the selection of proper ingredients, bioprocessing could moreover affect the nutritional and functional properties of GF bread. In particular, sourdough fermentation has largely been reported as a natural and effective biotechnology to improve the textural, sensorial, and nutritional properties as well as the shelf life of GF baked goods [19,20]. A proper selection of lactic acid bacteria (LAB) having specific metabolic traits able to affect the technological and the nutritional features of the product is strongly suggested [21]. Among all, the synthesis of exopolysaccharides (EPS) has recently gained attention due to the ability to counteract the negative effects associated with high levels of sourdough acidification and enhance the loaf volume [22]. EPS-producing strains belonging to the *Weissella* genus were widely investigated as potential starters to be employed in food fermentations. Indeed, the relevance of these heterofermentative LAB is related to their ability to grow at wide temperature, a_w_, and pH ranges [23] and to produce EPS that may improve the rheological properties of doughs [24].

This study aimed at optimizing a formulation and breadmaking procedure to obtain a novel “clean-label” GF bread. The formulation of the recipe was based on the selection of different ingredients naturally characterized by (i) high protein content, (ii) structuring properties, and (iii) high sucrose concentration, this latter able to promote the in situ EPS production by the selected *Weissella cibaria* P9. The bread made with the optimized production protocol was characterized for the main technological, nutritional, and sensory features.

## 2. Materials and Methods

### 2.1. Flours Selection

GF commercially available rice (*Oryza sativa*) (IPAFOOD, Frigento, Italy), psyllium (*Plantago psyllium*) (Biotiva, Straßlach-Dingharting, Germany), quinoa (*Chenopodium quinoa*) (Food For All, Settimo di Pescantina, Italy), chickpea (*Cicer arietinum*) (Effegi s.r.l., Spoleto, Italy), chestnut (*Castanea sativa* Miller) (Ki Group s.p.a., Torino, Italy), red lentil (*Lens culinaris*), pea (*Pisum sativum*), flaxseed (*Linum usitatissimum*), faba (*Vicia faba*); lupin (*Lupinus albus* L.,) (Il Fior di Loto s.r.l., Orbassano, Italy), wholemeal teff (*Eragrostis tef*), and corn (*Zea mays*) (EcorNaturaSì s.p.a., Verona, Italy) flours; and whole chia (*Salvia hispanica*) seeds (EcorNaturaSì s.p.a., Verona, Italy) were used in this study.

Prior to use, chia seeds were milled using a laboratory mill Ika-Werke M20 (GMBH, and Co. KG, Staufen, Germany). The proximate composition of flours is reported in Table 1. Sucrose in chestnut and quinoa flours was determined by Megazyme kits (Megazyme, Bray, Ireland) K-SUFRG following the manufacturer’s instructions.

#### 2.1.1. Selection of the Ingredients

Thanks to the widely proven suitability in GF bread [19], a blend of rice and corn flours in a ratio of 1:1 (*wt*/*wt*) was used here as the basic formula for breadmaking [25]. Aiming at increasing the protein content and improving the leavening capacity and the structural characteristics of the bread, the addition of protein-rich flours (>9% proteins) (quinoa, teff, chichpea, faba bean, lupin, red lentil and pea flours) and structuring agents-containing flours (psyllium, chia and flaxseed, below reported as “structuring flours”) (Table 1) to the basic formula was evaluated. Chia flour was used as such or after hydration, as previously described by Steffolani et al. [26].

Dough yield (DY, dough weight × 100/flour weight) was 210 for all the formulations and the level of fortification was 10% (*wt*/*wt*, on the flours total weight) for each protein-rich flour and 4% (*wt*/*wt*, on the flours total weight) for the structuring flours, when singly used. Mixtures of the psyllium flour with either chia or flaxseed (ratio 4:2), and both of them (4:1:1) were also used. When mixtures were used, the level of fortification of the structuring flours was 6% (*wt*/*wt*) on the flours’ total weight. All the recipes are reported in Supplementary Table S1. Baker’s compressed yeast (Lesaffre, Trecasali, Italy) at 1.25% (*wt*/*wt* final dough weight) was used according to the producer suggestion. Doughs (200 g) were mixed at 60× *g* for 5 min with an IM 5-8 high-speed mixer (Mecnosud, Flumeri, Italy), proofed at 30 °C for 2 h, and baked at 220 °C for 30 min (Combo 3, Zucchelli, Verona, Italy). Dough containing only rice and corn flours (ratio 1:1) was used as control (CT). Dough and bread were made in triplicate and analyzed twice.

#### 2.1.2. Evaluation of Leavening Capability and Structural Properties

The leavening capacity, by means of volume increase (ΔV, mL), was determined by measuring the dough volume at the end and beginning of the proofing and calculated as the differences between the values [27]. The leavening performance was expressed as the percentage of volume increase.

After baking, the loaves were cooled for 2 h before being weighed. Loaf volume was determined by the rapeseed displacement method AACC 10-05.01 [28]. The alveolus percentage of the bread crumb (ratio of the gas cell area on the total area of the bread slice) was evaluated after 24 h of storage using the image analysis technology. The UTHSCSA ImageTool software was used, as previously described by Rizzello et al. [29].

### 2.2. Sourdough Making and Characterization

#### 2.2.1. Sourdough Making

According to an abundant literature [7,22,30,31], the use of sourdough and EPS in the production of GF bread leads to several improvements in terms of structural and sensory properties as well as prolonged shelf life. However, the microbial synthesis of EPS is affected by the fermentable sugars, in particular from sucrose concentration [32]. According to the above consideration, Type-II sourdoughs (DY, 210) were prepared using a mixture of rice and corn flours (ratio, 1:1 *wt*/*wt*) as a basic recipe, quinoa and chestnut flours (at different level of fortification) as sucrose source, and *Weissella cibaria* P9 (previously isolated from pineapple and belonging to the culture collection of the Department of Soil, Plant and Food Sciences) as an EPS-producer strain [33]. Sourdough formulations are summarized in Table 2. Overall, 5 sourdoughs were prepared using either quinoa or chestnut flours at 10 and 20% (*wt*/*wt* on flours total weight) level of fortification. Moreover, a combination 1:1 of the two flours (20% *wt*/*wt* on the flours total weight) was also included (Supplementary Table S2). Cell suspensions were prepared as described by Rizzello et al. [34] and inoculated in doughs. The initial cell density of the lactic acid bacterium in dough was 7.0 log10 cfu/g. The fermentation was carried out at 30 °C for 24 h.

#### 2.2.2. Sourdough Characterization

Kinetics of the growth and acidification of *W. cibaria* P9 in the different sourdoughs were determined and modeled in agreement with the Gompertz equation, as modified by Zwietering et al. [35]. The experimental data were modeled by the non-linear regression procedure of the Statistica 12.0 software (Statsoft, Tulsa, OK, USA). The pH of doughs was determined by a M.507 pHmeter (Crison, Milan, Italy) equipped with a food penetration probe. Total Titratable Acidity (TTA) was determined after homogenization of 10 g of dough with 90 mL of distilled water and expressed as the amount (mL) of 0.1 M NaOH required to neutralize the solution to pH 8.4.

Water/salt-soluble extracts (WSE) were used to analyze organic acid, peptides, and free amino acids (FAA). Organic acids were determined by Megazyme kits (Megazyme, Bray, Ireland) K-DLATE and K-ACET (sum of D- and L- lactic and acetic acids, respectively) following the manufacturer’s instructions. The fermentation quotient (FQ) was determined as the molar ratio between lactic and acetic acids. Peptides concentration was determined by the *o*-phtaldialdehyde (OPA) method as described by Church et al. [36]. FAA were analyzed by a Biochrom 30+ series Amino Acid Analyzer (Biochrom Ltd., Cambridge Science Park, England) with a Li-cation-exchange column (20 by 0.46 cm inner diameter).

#### 2.2.3. Evaluation of EPS Production

The content of soluble dextran produced during fermentation by *W. cibaria* P9 was determined by an enzyme-assisted method using a mixture of dextranase (Sigma-Aldrich, Darmstadt, Germany) and α-glucosidase (Megazyme, Bray, Ireland) according to the method reported by Katina et al. [37]. Dextranase from *Chaetomium erraticum* and α-glucosidase from *Aspergillus niger* were purchased from Sigma-Aldrich (Germany). About 100 mg of freeze-dried dough was placed in 10 mL centrifuge tubes with 3 mL of water/ethanol (50:50 *v*/*v*) solution at 100 °C for 5 min. Another 3 mL of aqueous ethanol solution was added; then, the mixture was centrifuged at 10,000× *g* for 10 min. A further sample cleaning was made by re-suspending and centrifuging the pellet in 5 mL aqueous–ethanol solution. The pellet was re-suspended in 4.5 mL of sodium citrate buffer 0.05 M (pH 5.5) and kept at 100 °C for 5 min, vigorously vortexing after 2 min. The solutions cooled before the addition of a mixture of dextranase (100 nkat/mL) and α-glucosidase (10 nkat/mL), reaching the final volume of 5 mL with a hydrolysis temperature of 30 °C for 48 h. The reaction was stopped by keeping the samples in a boiling water bath for 10 min, after which the glucose formed was quantified using a K-GLUC glucose kit (Megazyme, Bray, Ireland).

### 2.3. Optimization of the Bread Formulation and Set-Up of the Breadmaking Protocol

#### 2.3.1. Formulation

According to the previous analysis, teff, lentil, and quinoa flours were selected as the best-performing high-protein flours, while the combination of psyllium, hydrated chia, and flaxseed (4:1:1) was the best mixture of structuring flours. The sourdough containing 20% (*wt*/*wt* on flours total weight) of chestnut flour was selected as the optimal compromise among EPS production and biochemical properties. Five different bread formulations were evaluated (Table 3).

All the breads contained rice and corn flours (1:1) and 6% (*wt*/*wt*) of the mixture (4:1:1) of psyllium, hydrated chia, and flaxseed. Teff, lentil, or quinoa flours were used at 10% (*wt*/*wt*) and sourdough inoculum was of 30% (*wt*/*wt*). Sourdough breads (DY 210) were manufactured at the pilot plant of the Department of Soil, Plant and Food Science of the University of Bari (Italy), according to the two-stage protocol including the production of sourdough (fermentation for 24 h at 30 °C, step I), and the mixing with flours (rice and corn), structuring agents, water, and compressed baker’s yeast, followed by incubation for 1.5 h at 30 °C (step II). Aiming at evaluating the effect of the protein-rich flours and sourdough on bread characteristics, a bread containing neither sourdough nor protein-rich flours (b-CT) and bread without protein-rich flours (b-S) were made and used as control. Baker’s yeast was added at the percentage of 1.25% (*wt*/*wt*) in all breads. Doughs were mixed at 60× *g* for 5 min with an IM 5-8 high-speed mixer (Mecnosud, Flumeri, Italy). All breads were baked at 210 °C for 30 min (Combo 3, Zucchelli, Verona, Italy).

#### 2.3.2. Sensory and Textural Characterization of the Experimental Breads

Sensory analysis of breads was carried out by 8 trained non-celiac panelists (4 male and 4 females, mean age: 32 years, range: 26–45 years). The training of panelists was conducted according to the method described by Elia [38]. The sensory attributes were discussed with the assessors during the introductory sensory training sessions. Before the sensory evaluation, the loaves were thawed at room temperature for 5–6 h and then cut into slices 1.5 cm thick. Each panelist received a piece per each sample. The color of crust and crumb, elasticity, crumbliness, acidic smell and taste, sweetness, bitterness, grassy (fresh-tasting notes of cut grass and herbs), and salty taste were the attributes used to describe different samples using a scale from 0 to10, with 10 being the highest score.

Texture profile analysis was performed by using an FRTS-100N Texture Analyzer (Imada, Toyohashi, Japan) equipped with a cylinder probe FR-HA-30J on boule-shaped loaves (200 g) stored for 24 h at room temperature after baking. The instrument settings were test speed 1 mm/s, 30% deformation of the sample, and two compression cycles, and the parameters evaluated were (i) hardness, (ii) springiness, and (iii) cohesiveness. The crumb features were determined as reported above. A commercial GF bread (Panini, BiAglut, Latina, Italy) was included in both analyses and used as control.

### 2.4. Characterization of the Selected “Clean-Label” GF Bread

According to the data collected, bread containing quinoa flour as an additional protein source, chestnut sourdough, and the mixture of the structuring flours was selected and characterized for proximate composition and nutritional features. The nutritional features of the experimental “clean-label” GF bread were compared to a reference dataset referring to commercial GF breads available in the Italian market [39].

#### 2.4.1. Proximate Composition

Protein (total nitrogen × 5.7), total lipids, ash, salt, and moisture contents were determined according to the AACC approved methods 46-11, 30-10.01, 08-01, 40-61.02, and 44-15A, respectively [28], while the determination of total dietary fibers was carried out by AOAC-approved methods 991.42 [40]. Total available carbohydrates were calculated as the difference [100 − (moisture + proteins + lipids + ash + total dietary fibers)] [41]. Saturated lipids were analyzed by Enotecnolab (Conversano, Italy). Proteins, lipids, carbohydrates, and ash were expressed as percentage of total weight.

#### 2.4.2. Protein Digestibility, Starch Hydrolysis, and Predicted Glycemic Index

The in vitro protein digestibility (IVPD) of the bread was determined by the method of Akeson and Stahmann [42]. One gram of sample was incubated with 1.5 mg of pepsin, in 15 mL of 0.1 M HCl, at 37 °C for 3 h. After neutralization with 2 M NaOH and addition of 4 mg of pancreatin, in 7.5 mL of phosphate buffer (pH 8.0), 1 mL of toluene was added to prevent microbial growth, and the solution was incubated at 37 °C for 24 h. After 24 h, the enzyme was inactivated by the addition of 10 mL of trichloroacetic acid (20%, *wt*/*v*), and the undigested protein was precipitated. The volume was made up to 100 mL with distilled water and centrifuged at 5000 rpm for 20 min. The precipitate was subjected to protein extraction, according to Weiss et al. [43], and the protein concentration in the solutions was determined by Bradford method [44]. The IVPD was expressed as the percentage of the total protein, which was solubilized after enzyme hydrolysis.

The analysis of starch hydrolysis, mimicking the in vivo digestion of starch [45], was carried out in a portion, containing 1 g of starch, of bread made using the final formulation of bread. The glucose content was measured with a D-Fructose/D-Glucose assay kit (Megazyme, Bray, Ireland). The degree of starch digestion was expressed as the percentage of potentially available starch hydrolyzed at different times (30, 60, 90, 120, 150, and 180 min). A non-linear model [45] was applied to describe the kinetics of starch hydrolysis obtaining a curve following the first-order equation: C = C∞ (1 – e − kt) where C is the concentration at t time, C∞ is the equilibrium concentration, k is the kinetic constant, and t is the chosen time. Wheat flour bread (WB) was used as the control to estimate the hydrolysis index (HI = 100). The predicted GI was calculated using the equation: GI = 0.549 × HI + 39.71 [46] with wheat bread as the reference (GI wheat bread = 100).

### 2.5. Statistical Analysis

Data were subjected to one-way ANOVA; pair-comparison of treatment means was obtained by Tukey’s procedure at *p* < 0.05, using the statistical software Statistica 12.0 (StatSoft Inc., Tulsa, OK, USA).

## 3. Results

### 3.1. Flour Selection

**Figure 1:**
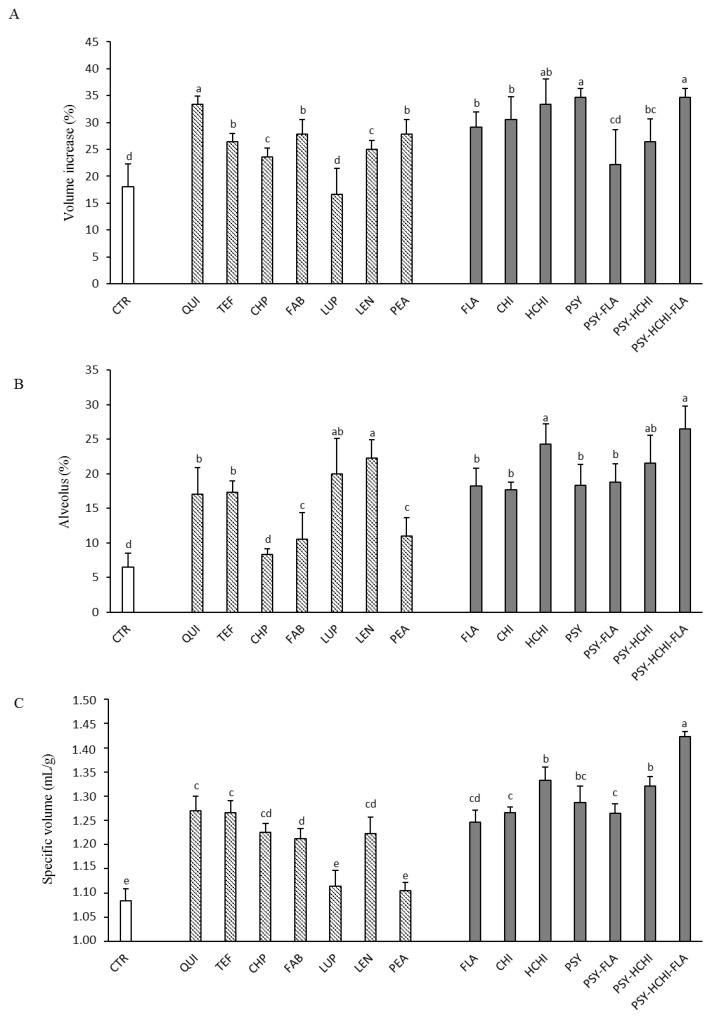
Evaluation of leavening performances on dough and bread made with rice and corn flours and supplemented with 10% of protein-rich (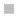) and structuring (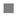) flours (QUI, quinoa; TEF, teff; CHP, chickpea; FAB faba bean; LUP, lupin; LEN, lentil; PEA, pea; FLA, flaxseed; CHI, chia; HCHI, hydrated chia; PSY, psyllium). (**A**): Volume increment of dough after leavening (2 h at 30 °C); (**B**): Alveolus percentage of bread slice; (**C**): Specific volume of bread. ^a–e^ Bars with different letters differ significantly (*p* < 0.05)

The influence of the fortification of the rice–corn blend with protein-rich and structuring flours on the technological properties of dough and bread was evaluated by means of the volume increase of dough after leavening, the specific volume of the loaf, and the alveolus percentage of the bread slices, respectively (Figure 1A–C).

Among protein-rich flours, quinoa led to the highest volume increase of dough (+33.3 ± 1.6%) and the highest specific volume of bread (1.27 ± 0.03 mL/g), while the highest alveolus percentage was observed when lentil flour was added to the bread recipe (22.2 ± 2.7%). No significant (*p* < 0.05) differences were found in alveolus percentage and specific volume of bread when quinoa or teff were used, while the addition of the latter one led to a significantly lower volume increase of the dough (26.4 ± 1.6%) (Figure 1A). Conversely, when lupin flour was added, similar results to control dough and bread (made using only rice and corn flours) were found in terms of volume increase of dough and specific volume of bread. Moreover, no significant differences were found in alveolus percentage of control bread and chickpea-fortified bread, and specific volume of control bread and pea-fortified bread. Regarding the structuring flours, all (singly or mixed) positively affected the specific volume of bread; however chia, hydrated chia, and the mixture 4:1:1 (psyllium: hydrated chia: flaxseed) corresponded to the best results in terms of volume increment of dough, alveolus percentage of bread slice, and specific volume of bread, compared to the others. When structuring flours were added singly, hydrated chia led to better results for all the parameters considered: (i) volume increase (+33.3 ± 4.8%), (ii) specific volume (1.33 ± 0.03 mL/g), and (iii) alveolus percentage (24.3 ± 2.9%) with no significant (*p* < 0.05) difference in leavening capability and specific volume compared to psyllium flour fortification. However, the combination of structuring flours (4:1:1) led to the best results: (i) volume increase (+34.7 ± 1.6%), (ii) alveolus percentage (26.4 ± 3.3%), and (iii) specific volume of bread (1.42 ± 0.02 mL/g) (Figure 1A–C).

According to these results, quinoa, teff, or lentil flours, and the mix of psyllium (4% *wt*/*wt*), hydrated chia (1% *wt*/*wt*), and flaxseed (1% *wt*/*wt*) were selected for further analysis.

### 3.2. Sourdough Characterization and EPS Production

Chestnut and quinoa flours were used as sucrose sources to promote the synthesis of EPS by *W. cibaria* P9 during sourdough fermentation. Their total sugars content corresponded to 26.6% and 6.0%, respectively, while sucrose concentration was 15.22 ± 0.27% (of dry matter) in chestnut and 2.95 ± 0.12% (of dry matter) in quinoa. The mathematical modulation of cell density during fermentation showed the lowest µmax value in rice–corn sourdough (s-CT, 0.65 ± 0.03 Δlog cfu/h). A significant higher µmax was found when alternative flours were used; however, the highest values were found in s-Q10 and s-Q20 (1.16 ± 0.06 and 0.98 ± 0.07 Δlog cfu/h, respectively). The shortest lag time was found for s-C20, s-CT, and s-CQ10 (*circa* 1.81 h). Conversely, the acidification kinetics showed the longer lag time in s-C10 and s-CT, while the shortest ones were detected in s-CQ10 and s-C20 (0.84 ± 0.05 and 1.04 ± 0.05 h, respectively). The pH before fermentation (T0) ranged from 5.81 ± 0.03 (s-C20) to 6.01 ± 0.06 (s-CT), and it decreased during the fermentation with pH values lower than 4.3 in all the sourdoughs. The highest and lowest pH were found in s-C20 (4.28 ± 0.05) and s-CT (3.94 ± 0.08), respectively after 24 h of incubation (T24) (Table 4).

TTA was positively correlated with the inclusion of alternative flour before (r = 0.79, *p* < 0.001) and after (r = 0.96, *p* < 0.001) fermentation. The inclusion of quinoa or chestnut flours led to the highest increase of lactic or acetic acids, respectively. Therefore, lower FQ were found in s-C20 (2.19) as compared to s-Q20 (4.50). Moreover, the lowest FQ was found in s-CQ10 due to the lower content of lactic acid (69.3 ± 1.4 mmol/kg). The use of protein-rich and structuring flours led to a higher concentration of peptides and TFAA in doughs prior to fermentation (T_0_). The concentration of TFAA and peptides up to 5.6- and 2-folds higher were found in fortified sourdough (T_0_) as compared to s-CT (T_0_). Moreover, a more intense proteolysis occurred in sourdoughs fortified with the alternative flours compared to s-CT. Indeed, values up to 2 g/kg (peptides) and 1 g/kg (TFAA) higher were found in fortified sourdoughs (T_24_) as compared to s-CT (T_24_), and the highest values were detected in s-Q20 (Table 4).

As expected, the sucrose provided with quinoa and chestnut flours supplementation led to a higher content of EPS after fermentation, which was mainly represented by dextran. The highest amount was detected in s-C20 (0.65 ± 0.06 g/kg), while no significant differences were found between s-C10 and s-Q20 (0.40 ± 0.03 and 0.43 ± 0.02 g/kg, respectively). Only 0.07 ± 0.02 g/kg of dextran were detected in the control s-CT.

### 3.3. Sensory Analysis and Structural Properties

Sensory analysis was performed to evaluate the impact of (i) structuring flours, (ii) protein-rich flours, and (iii) sourdough on the organoleptic characteristics of bread. Overall, the use of sourdough significantly changed the sensory profile of breads. As expected, compared to b-CT, higher values of elasticity, saltiness, color of crust and crumb, acidic smell, and taste were perceived by the panelists when sourdough was included in the formulation. Although the acidic taste decreased, a further increase of the saltiness, crust, and crumb color was found with the use of protein-rich flours, as compared to b-S (Table 5). Moreover, the highest value of salty and grassy tastes (5.2 ± 0.4 and 4.2 ± 0.7) were detected when teff was used.

The instrumental analysis of texture showed a decrease of hardness when bread loafs were made using sourdough (Table 5) and the inclusion of protein-rich flours led to a further decrease, with the lowest value found in b-SL (Table 5). Conversely, springiness, which represents the rate at which a deformed material returns to the undeformed condition after deforming force is removed, increased. The cohesiveness, which represent the strength of the internal bonds making up the body of the product, was found similar in all breads. The only statistical differences (*p* < 0.05) were found comparing b-ST with b-SQ, b-SL, and b-CGF, representing the negative effect, for this parameter, of teff flour inclusion. No statistical differences were found for b-CT and b-S when compared with other breads.

**Figure 2:**
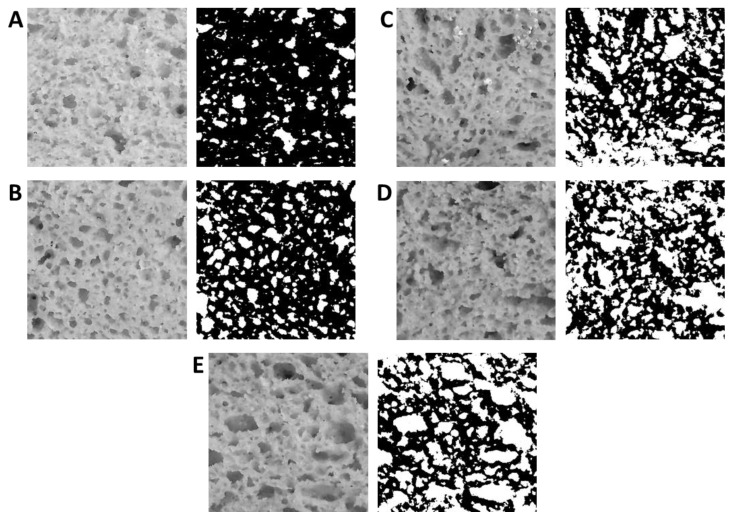
Representative images including digital images of bread showing the original gray-level images and computed binary results from gray-level thresholding at the two-cluster of bread crumb. b-CT (**A**), bread made with rice and corn flours (ratio 1:1) and 6% (*wt*/*wt*) of structuring flours (psyllium, hydrated chia, and flaxseed, ratio 4:1:1); b-S (**B**), bread made with rice and corn flours (ratio 1:1), 30% (*wt*/*wt*) of s-C20, and 6% of structuring flours (psyllium, hydrated chia, and flaxseed, ratio 4:1:1); b-ST (**C**), bread made with rice and corn flours (ratio 1:1), 30% (*wt*/*wt*) of s-C20, 10% (*wt*/*wt*) of teff flour, and 6% of structuring flours (psyllium, hydrated chia, and flaxseed, ratio 4:1:1); b-SL (**D**), bread made with rice and corn flours (ratio 1:1), 30% (*wt*/*wt*) of s-C20, 10% (*wt*/*wt*) of red lentil flour, and 6% of structuring flours (psyllium, hydrated chia, and flaxseed, ratio 4:1:1); b-SQ (**E**), bread made with rice and corn flours (ratio 1:1), 30% (*wt*/*wt*) of s-C20, 10% (*wt*/*wt*) of quinoa flour, and 6% of structuring flours (psyllium, hydrated chia, and flaxseed, ratio 4:1:1). All doughs had DY of 200. Baker’s yeast was used at 1.25% (*wt*/*wt*). Proofing was at 30 °C for 2 h and baking at 200 °C for 45 min.

The image analysis of bread crumb showed the increase of alveolus percentage in all experimental breads, as compared to b-CT (14.59 ± 0.43) (Figure 2A). Indeed, the inclusion of sourdough in bread formulation led to higher gas retention during leavening with a significant (*p* < 0.05) increase of white spots, which were doubled in b-S (30.66 ± 4.18) and tripled in bread made using protein-rich flour (from 48.17 ± 1.94 to 48.82 ± 3.53).

Therefore, quinoa flour was chosen to increase the protein content of the final recipe without affecting the structural and sensorial value of the experimental bread.

### 3.4. Nutritional Characterization of Bread

The proximate composition and nutritional features of the bread made by using the new biotechnological protocol were investigated; results are reported in Table 6. The experimental bread was characterized by higher moisture (43.6 ± 2.4%) and carbohydrates (47.0 ± 1.7 g/100 g) content compared to the reference dataset, with only 0.6 ± 0.2 g/100 g represented by sugar. The amount of lipids was very low (1.8 ± 0.4 g/100 g), and only 0.3 ± 0.1 g/100 g was represented by saturated ones. Dietary fibers concentration was lower than the median of commercial counterparts. The protein content of bread was 5.0 ± 0.2 g/100 g representing 8.7% of the total calories provided by bread. This value represents a protein content two-fold higher than the reference value characterizing commercial products [39]. No additional salt was included in the final bread formulation; therefore, the sodium amount corresponded to that naturally present in flours.

The IVPD of the experimental bread was *circa* 77%, while a median value of *circa* 21% characterized commercial GF products. Moreover, the predicted glycemic index, which was calculated evaluating starch hydrolysis kinetics during 3 h of mimicked digestion, was 85 ± 3.1 (Table 6).

## 4. Discussion

In addition to celiac patients, the increasing consumer demand for products with “reduced” or “no gluten” content categories to face gluten sensitivity and lactose intolerance disturbances requires the innovation and differentiation of gluten-free products [47]. Moreover, the amount of attention on the “free-from” foods, mainly because consumers consider them healthier [48], is also forcing the industries toward the production of “clean-label” products. Although, a unique definition of this term as well as the features that a “clean label” food should highlight are still debated [10], the preparation of a “clean label” GF bread should avoid the use of chemical additives [10], which in turn are usually included in such bread formula. Indeed, although the technological process allowed acquiring significant achievements in the overall quality of the gluten-free products, the absence of gluten still represents an industrial challenge to produce baked goods [49]. Moreover, concerns about the nutritional composition of gluten-free foods are often reported [50]. To face nutritional deficiencies, the inclusion of a protein source in GF bread is a consolidate strategy; nevertheless, the final protein content tends to be lower than that of wheat-containing counterparts [50,51]. Overall, plant-derived proteins are preferred to animal protein sources (e.g., egg and dairy products) because, thanks to their better water-retention capacity, they exert positive effects on the texture of GF bread [52].

Sourdough technology has widely been used to improve the physicochemical, sensory, and nutritional attributes of GF bread [53]. Indeed, different GF cereals such as sorghum, millet, rice, corn, and buckwheat have been investigated as ingredients to develop GF sourdough breads [53,54,55,56].

The positive effects of sourdough are mainly due to the metabolic activities of the lactic acid bacteria performing the fermentation, and recently, the use of *Weissella* species as novel starter cultures for sourdough bread production has been reported by several authors [57,58,59,60].

In this framework, the present study aimed at optimizing a GF bread formulation including not-refined plant-based flours as a source of protein and structuring ingredients. The use of a Type-II sourdough obtained through *Weissella cibaria* P9 fermentation, an EPS-producing strain, has also been evaluated. The effect of the ingredients and sourdough fermentation on the main structural, sensory, and nutritional properties of the bread has been investigated.

A preliminary evaluation of the effect of protein-rich flours on the dough leavening performance (volume increase) and bread structural properties (specific volume and alveolus percentage) has shown a positive influence of quinoa, teff, and lentil flours. The inclusion of quinoa and teff has already been reported as effective in improving the leavening capability of GF bread [61,62], such as the nutritional and sensorial quality of GF bread [14,63]. Moreover, Asif and colleagues demonstrated that lentil protein isolates possess good foaming and emulsifying properties [64]. The effects of teff, quinoa, and lentil flours on the sensory profile of bread were also assessed. The inclusion of quinoa led to the enhancement of crust and crumb color, as previously reported by Turkut and colleagues [62], meeting the consumer concerns about the common light color of GF bread [65]. Moreover, the inclusion of 20–25% of quinoa flour was already proposed by several authors as a reliable tool to improve the sensory acceptability of conventional and GF breads [39,66].

The natural structuring ingredients chia (raw and hydrated), flaxseed, and psyllium flours were tested singly and as a mixture to evaluate the technological effect in GF bread formulation. Studies on commercial GF breads revealed the use of a combination of several starchy sources or gluten replacers to optimize bread quality [9]. Despite the increasing inclusion in GF commercial bread, very few research studies incorporate psyllium, and no study evaluated psyllium-based formulas including other natural structuring ingredients [9,67]. In this work, the supplementation of all the structuring flours improved the volume increase and the structural properties when singly used, as already reported elsewhere [26,67,68,69,70]. However, when psyllium, hydrated chia, and flaxseed were combined, a further enhancement of the textural quality of dough and bread was achieved.

Aiming at setting up a “clean-label” product, EPS were produced in situ by using *W. cibaria* P9 on matrices naturally containing sucrose. Although belonging to the natural microbiota of several fermented food products [71], *Weissella* species have not yet been included in the Qualified Presumption of Safety (QPS) list of European Food Safety Authority (EFSA) due to the safety concerns [71,72]. However, the rare and opportunistic features of the infection cases related to *Weissella* strains have recently been highlighted, and its species been proposed to be granted the QPS status [72].

EPS display physiochemical properties similar to commercial purified hydrocolloids, allowing the improvement of bread structure and loaf volume, enhancing crumb softness and delaying bread staling [22]. Moreover, dextrans produced by *W. cibaria* were recently associated with a positive modulation of gut microbiota and influence on body weight and metabolism [73]. The in situ formation of EPS (homo-polysaccharides) in sourdough was reported to be more effective than external addition [30] and does not require labeling; thus, this strengthens their case for use in “clean-label” products [74].

As expected, the higher the content of the sucrose in the dough, the higher the EPS content at the end of the fermentation. Although the highest percentage of chestnut flours to avoid sensory or technological adverse effects was used [47], a low amount of EPS was produced during sourdough fermentation. The relatively low amount of sucrose in the dough and the short fermentation time [75,76] were reported as responsible for the poor EPS synthesis during fermentation [77,78]. However, the role of texture improvers was, in the proposed formulation, synergistic among different components, including the different structuring flours.

The final formulation of GF bread, including (i) quinoa as a protein source, (ii) the mixture of psyllium, hydrated chia, and flaxseed as a structuring (4:1:1) agent, and (iii) the chestnut sourdough containing EPS as improver, was characterized by a protein content that was more than 50% higher than a GF product usually found in the market [39,50], and it also had a high value of in vitro protein digestibility (76.9 ± 2.7) [39,51]. Moreover, the optimized bread formula contained a low amount of lipids, which were mainly represented by unsaturated fatty acids, previously characterized by an optimal n-6/n-3 polyunsaturated fatty acids ratio and recognized as carriers of nutraceutical components such as tocopherols and sterols [18,79,80]. Finally, the low sugar content of bread led to a similar in vitro glycemic index as compared to commercial GF bread [39,81].

## 5. Conclusions

Here, the formulation of the novel “clean-label” GF bread has been optimized by using (i) a mixture of corn and rice flour (ratio 1:1); (ii) quinoa flour (10%) as a source of protein, and (iii) psyllium, flaxseed, and hydrated chia (6%) as structuring agents. Moreover, a type-II sourdough, which was obtained by using a selected *Weissella cibaria* P9 and chestnuts flour as a sucrose source to promote the in situ exo-polysaccharides syntheses, was included (30% of the final dough) in the bread formulation. Overall, the novel GF bread was characterized by good textural properties, high protein content (8.9% of dry matter) and in vitro protein digestibility (76.9%), low sugar (1.0% of dry matter) and fat (3.1% of dry matter) content, and an in vitro predicted glycemic index of 85. Moreover, common sourdough bread features, i.e., acidic taste and darker color of both crust and crumb, were identified in the “clean-label” GF bread. This work demonstrates that a proper selection of raw materials (unprocessed flours), together with the use of traditionally inspired biotechnology, allow the production of high-quality bread, meeting the demand of the modern consumer for novel clean-label GF products.

## Figures and Tables

**Table 1:** Proximate composition (percentage) of the flours used in this study as reported on the commercial labels.

	Energy Value (kJ/100 g)	Moisture	Total Carbohydrates	Sugars	Dietary Fibers	Lipids	Saturated Lipids	Proteins	Salt
Rice	1478	10.6	80.0	1.2	0.5	0.6	0.1	7.0	0.01
Corn	1449	12.9	73.0	0.9	4.5	1.3	0.2	7.2	n.d.
Chestnut	1527	11.0	69.2	26.6	10.5	3.6	0.6	5.6	0.03
Quinoa	1632	12.1	66.0	6.0	3.8	5.7	1.7	12.4	0.02
Teff	1431	14.6	65.2	1.5	7.6	3.2	0.6	9.4	0.01
Chickpea	1402	10.1	51.5	3.5	13.1	4.6	0.6	20.7	0.02
Faba	1570	10.1	53.8	4.8	6.8	2.9	0.5	26.4	n.d.
Lupin	1.519	11.0	18.2	2.9	29.0	6.4	1.9	35.4	0.03
Red lentil	1477	12.0	50.6	2.2	12.9	0.3	0.1	24.2	0.01
Pea	1427	11.0	48.2	2.8	20.3	1.0	n.d.	19.5	0.01
Psyllium	779	11.8	1.6	n.d.	85.0	0.6	0.1	1.6	0.10
Flaxseed	2196	10.3	1.7	1.6	23.0	42.0	3.4	23.0	0.07
Chia	1856	11.7	1.6	0.9	31.9	32.1	3.6	22.7	0.01

**Table 2:** Formulations of the experimental sourdough (DY = 210) prepared using rice and corn four substituted from 10 to 20% with quinoa and chestnut flours. Sourdoughs were fermented by *Weissella cibaria* P9 (initial cell density in dough of 7.0 log10 cfu/g) at 30 °C for 24 h.

	s-CT	s-C10	s-C20	s-Q10	s-Q20	s-CQ10
Rice	23.81	21.43	19.05	21.43	19.05	19.05
Corn	23.81	21.43	19.05	21.43	19.05	19.05
Chestnut	n.i.	4.76	9.52	n.i.	n.i.	4.76
Quinoa	n.i.	n.i.	n.i.	4.76	9.52	4.76
Water	52.38	52.38	52.38	52.38	52.38	52.38

Data are expressed as percentage (%) of total dough weight. n.i., not included.

**Table 3:** Formulations of the experimental breads. Sourdough (DY = 210) was prepared using rice, corn, and chestnut flours (4:4:2). Fermentation by *Weissella cibaria* P9 at 30 °C lasted 24 h. Baker’s yeast was used at 1.25% (*wt*/*wt*).

	b-CT	b-S	b-SQ	b-ST	b-SL
Rice	22.38	15.24	12.86	12.86	12.86
Corn	22.38	15.24	12.86	12.86	12.86
Quinoa	n.i.	n.i.	4.76	n.i.	n.i.
Teff	n.i.	n.i.	n.i.	4.76	n.i.
Lentil	n.i.	n.i.	n.i.	n.i.	4.76
Sourdough	n.i.	30.00	30.00	30.00	30.00
Psyllium	1.90	1.90	1.90	1.90	1.90
Hydrated chia *	1.90	1.90	1.90	1.90	1.90
Flaxseed	0.48	0.48	0.48	0.48	0.48
Water	50.96	35.25	35.25	35.25	35.25

Data are expressed as percentage (%) of total dough weight. * The hydration process (chia flour: tap water ratio, 1:3) was carried out for 30 min at 20 °C. n.i., not included.

**Table 4:** Chemical evaluation of sourdoughs.

	s-CT	s-Q10	s-Q20	s-C10	s-C20	sCQ10
pH T_0_	6.01 ± 0.06 ^a^	5.97 ± 0.05 ^a^	5.94 ± 0.08 ^a^	5.86 ± 0.05 ^ab^	5.81 ± 0.03 ^b^	5.89 ± 0.04 ^ab^
pH T_24_	3.94 ± 0.08 ^d^	4.03 ± 0.06 ^cd^	4.13 ± 0.06 ^b^	4.11 ± 0.06 ^bc^	4.28 ± 0.05 ^a^	4.17 ± 0.04 ^b^
TTA T_0_ (mL NaOH 0.1 M)	1.4 ± 0.1 ^c^	2.0 ± 0.2 ^b^	2.0 ± 0.1 ^b^	1.8 ± 0.1 ^b^	2.8 ± 0.2 ^a^	2.8 ± 0.1 ^a^
TTA T_24_ (mL NaOH 0.1 M)	6.2 ± 0.2 ^e^	7.0 ± 0.3 ^d^	8.6 ± 0.2 ^b^	7.6 ± 0.3 ^c^	8.5 ± 0.2 ^b^	9.0 ± 0.1 ^a^
Peptides T_0_ (mg/kg)	915 ± 33 ^d^	1328 ± 71 ^b^	1401 ± 82 ^b^	1231 ± 16 ^c^	1875 ± 40 ^a^	1553 ± 78 ^b^
Peptides T_24_ (mg/kg)	1021 ± 11 ^e^	1354 ± 98 ^d^	3199 ± 79 ^a^	1875 ± 54 ^c^	2052 ± 29 ^b^	1397 ± 26 ^d^
TFAA T_0_ (mg/kg)	281 ± 11 ^f^	413 ± 9 ^e^	555 ± 10 ^d^	918 ± 7 ^c^	1593 ± 14 ^a^	1098 ± 16 ^b^
TFAA T_24_ (mg/kg)	1648 ± 18 ^d^	1609 ± 16 ^e^	2600 ± 13 ^a^	1774 ± 11 ^c^	2108 ± 21 ^b^	1648 ± 14 ^d^
Acetic acid (mmol/kg)	36.8 ± 0.8 ^c^	37.2 ± 0.7 ^bc^	30.4 ± 0.8 ^d^	38.4 ± 0.8 ^ab^	39.7 ± 1.1 ^a^	39.3 ± 1.0 ^a^
Lactic acid (mmol/kg)	91.9 ± 2.2 ^c^	115.3 ± 1.5 ^b^	136.8 ± 1.8 ^a^	95.2 ± 2.1 ^c^	86.9 ± 2.3 ^d^	69.3 ± 1.4 ^e^
FQ	2.50	3.10	4.50	2.48	2.19	1.77
Dextran (g/kg)	0.07 ± 0.02 ^e^	0.21 ± 0.03 ^d^	0.43 ± 0.02 ^b^	0.40 ± 0.03 ^b^	0.65 ± 0.06 ^a^	0.32 ± 0.04 ^c^

s-CT, sourdough made with rice and corn flours (ratio 1:1); s-Q10, sourdough made with rice and corn flours (ratio 1:1), and 10% (*wt*/*wt*, on flours total weight) of quinoa flour; s-Q20, sourdough made with rice and corn flours (ratio 1:1), and 20% (*wt*/*wt*, flours total weight) of quinoa flour; s-C10, sourdough made with rice and corn flours (ratio 1:1), and 10% (*wt*/*wt*, on flours total weight) of chestnut flour; s-C20, sourdough made with rice and corn flours (ratio 1:1), and 20% (*wt*/*wt*, on flours total weight) of chestnut flour; s-QC10, sourdough made with rice and corn flours (ratio 1:1), 10% (*wt*/*wt*, on flours total weight) of quinoa flour, and 10% (*wt*/*wt*, on flours total weight) of chestnut flour. Fermentation was carried out with *Weissella cibaria* P9 (initial cell density of 7 log10 cfu/g) at 30 °C for 24 h. All sourdoughs had DY of 210. TTA, Total Titratable Acidity; TFAA, Total Free Amino Acids; FQ, Fermentation Quotient. ^a–e^ Values in the same row with different superscript letters differ significantly (*p* < 0.05).

**Table 5:** Sensory and texture profile analysis of breads.

	b-CT	b-S	b-SQ	b-ST	b-SL	b-CGF
Crust colour	2.5 ± 0.4 ^c^	4.7 ± 0.7 ^b^	6.3 ± 0.6 ^a^	4.5 ± 0.8 ^b^	5.7 ± 0.8 ^ab^	2.7 ± 0.8 ^ab^
Crumb colour	2.9 ± 0.3 ^b^	5.3 ± 0.6 ^a^	6.1 ± 0.6 ^a^	5.5 ± 0.7 ^a^	5.6 ± 0.7 ^a^	1.6 ± 0.7 ^a^
Elasticity	6.1 ± 0.8 ^c^	7.7 ± 0.4 ^ab^	8.3 ± 0.4 ^a^	7.5 ± 0.8 ^b^	7.0 ± 0.6 ^bc^	3.0 ± 0.6 ^bc^
Crumbliness	3.9 ± 0.5 ^a^	3.8 ± 0.9 ^a^	3.3 ± 0.8 ^ab^	2.5 ± 0.8 ^b^	3.3 ± 0.9 ^ab^	3.3 ± 0.9 ^ab^
Acid smell	0.6 ± 0.4 ^c^	3.8 ± 0.7 ^ab^	3.5 ± 0.7 ^ab^	3.3 ± 0.5 ^b^	4.3 ± 0.7 ^a^	1.3 ± 0.7 ^a^
Acid taste	0.5 ± 0.3 ^c^	5.2 ± 0.7 ^a^	4.2 ± 0.3 ^b^	4.3 ± 0.7 ^ab^	4.4 ± 0.3 ^ab^	1.4 ± 0.3 ^ab^
Sweetness	5.6 ± 0.6 ^a^	3.1 ± 0.7 ^b^	3.7 ± 0.6 ^b^	4.0 ± 0.7 ^b^	3.7 ± 0.8 ^b^	2.7 ± 0.8 ^b^
Bitterness	2.3 ± 0.7 ^ab^	2.8 ± 0.7 ^a^	1.8 ± 0.6 ^b^	1.8 ± 0.5 ^b^	2.7 ± 0.8 ^a^	2.4 ± 0.8 ^a^
Grassy	1.1 ± 0.5 ^c^	2.7 ± 0.7 ^b^	2.2 ± 0.5 ^b^	4.2 ± 0.7 ^a^	1.5 ± 0.4 ^c^	0.5 ± 0.4 ^c^
Salty	2.7 ± 0.3 ^c^	4.0 ± 0.5 ^b^	4.5 ± 0.4 ^b^	5.2 ± 0.4 ^a^	4.2 ± 0.2 ^b^	5.1 ± 0.2 ^b^
Hardness (N)	59.06 ± 2.45 ^a^	46.47 ± 2.74 ^b^	39.56 ± 2.68 ^c^	39.16 ± 2.81 ^c^	33.75 ± 1.87 ^d^	32.34 ± 1.59 ^d^
Cohesiveness	0.267 ± 0.038 ^ab^	0.249 ± 0.025 ^ab^	0.272 ± 0.024 ^a^	0.238 ± 0.019 ^b^	0.277 ± 0.026 ^a^	0.279 ± 0.018 ^a^
Springiness	0.544 ± 0.114 ^b^	0.760 ± 0.087 ^a^	0.779 ± 0.107 ^a^	0.836 ± 0.098 ^a^	0.713 ± 0.094 ^a^	0.886 ± 0.097 ^a^

b-CT, bread made with rice and corn flours (ratio 1:1) and 6% (*wt*/*wt*) of structuring flours (psyllium, hydrated chia, and flaxseed, ratio 4:1:1); b-S, bread made with rice and corn flours (ratio 1:1), 30% (*wt*/*wt*) of s-C20, and 6% of structuring flours (psyllium, hydrated chia, and flaxseed, ratio 4:1:1); b-SQ, bread made with rice and corn flours (ratio 1:1), 30% (*wt*/*wt*) of s-C20, 10% (*wt*/*wt*) of quinoa flour, and 6% of structuring flours (psyllium, hydrated chia, and flaxseed, ratio 2:1:1); b-ST, bread made with rice and corn flours (ratio 1:1), 30% (*wt*/*wt*) of s-C20, 10% (*wt*/*wt*) of teff flour, and 6% of structuring flours (psyllium, hydrated chia, and flaxseed, ratio 4:1:1); b-SL, bread made with rice and corn flours (ratio 1:1), 30% (*wt*/*wt*) of s-C20, 10% (*wt*/*wt*) of red lentil flour, and 6% of structuring flours (psyllium, hydrated chia, and flaxseed, ratio 4:1:1). All doughs had DY of 200. Baker’s yeast was used at 1.25% (*wt*/*wt*). Proofing was at 30 °C for 2 h and baking at 200 °C for 45 min. b-CGF, a commercial gluten free bread (Panini, BiAglut, Latina, Italy) was included in the analyses and used as control. ^a–d^ Values in the same row with different superscript letters differ significantly (*p* < 0.05).

**Table 6:** Proximate composition and nutritional evaluation of the experimental “clean-label” gluten-free (GF) bread.

	Experimental Bread	Reference *
Energy (kJ)	962 ± 45	1032
Moisture (%)	43.6 ± 2.4	37.0
Total carbohydrates (g/100 g)	47.0 ± 1.7	45.9
Sugar (g/100 g)	0.6 ± 0.2	1.9
Dietary fiber (g/100 g)	1.7 ± 0.3	8.8
Total lipids (g/100 g)	1.8 ± 0.4	3.8
Saturated lipids (g/100 g)	0.3 ± 0.1	1.0
Protein (g/100 g)	5.0 ± 0.2	2.7
Salt (g/100 g)	0.1 ± 0.1	1.6
In vitro protein digestibility (%)	76.9 ± 2.7	21.17
Predicted glycemic index	85.0 ± 3.1	81.5

Results were compared to a reference dataset referring to commercial gluten-free breads available in the Italian market reported by Rizzello and colleagues [39]. * Data correspond to the median values of the distribution of the results obtained for all the commercial products [39].

## Data Availability

Not applicable.

## Supplementary Materials

The following are available online at https://www.mdpi.com/2304-8158/10/2/462/s1. Table S1: Formulations of the experimental doughs (DY = 210) made using protein-rich or structuring flours. Baker’s yeast was used at 1.25% (*wt*/*wt*). Table S2. Formulations of the experimental sourdoughs (DY = 210) made with rice and corn flours, and supplemented with quinoa and chestnut flours (10 or 20% of the flours total weight). Sourdoughs were fermented by *Weissella cibaria* P9 (initial cell density in dough of 7.0 log10 cfu/g) at 30 °C for 24 h.
