# Supplementary material for: Design of a “Clean-Label” Gluten-Free Bread to Meet Consumers Demand
Source: Foods. 2021 Feb 20;10(2):462. doi: 10.3390/foods10020462 (PMC7923426; doi:10.3390/foods10020462)
Supplement: Supplementary file 1 [file foods-10-00462-s001.pdf]

Supplementary Table 1. Formulations of the experimental doughs (DY = 210) made using protein-rich or structuring flours. Baker's yeast was used at 1.25 % (wt/wt).

	**CTR**	**QUI**	**TEF**	**CHP**	**FAB**	**LUP**	**LEN**	**PEA**	**PSY**	**CHI**	**HCHI**	**FLE**	**PSY-FLE**	**PSY-HCHI**	**PSY-HCHI-FLE**
Rice	23.81	21.43	21.43	21.43	21.43	21.43	21.43	21.43	22.86	22.86	22.86	22.86	22.38	22.38	22.38
Corn	23.81	21.43	21.43	21.43	21.43	21.43	21.43	21.43	22.86	22.86	22.86	22.86	22.38	22.38	22.38
Quinoa	-	4.76	-	-	-	-	-	-	-	-	-	-	-	-	-
Teff	-	-	4.76	-	-	-	-	-	-	-	-	-	-	-	-
Chickpea	-	-	-	4.76	-	-	-	-	-	-	-	-	-	-	-
Faba	-	-	-	-	4.76	-	-	-	-	-	-	-	-	-	-
Lupin	-	-	-	-	-	4.76	-	-	-	-	-	-	-	-	-
Red lentil	-	-	-	-	-	-	4.76	-	-	-	-	-	-	-	-
Pea	-	-	-	-	-	-	-	4.76	-	-	-	-	-	-	-
Psyllium	-	-	-	-	-	-	-		1.90	-	-	-	1.90	1.90	1.90
Chia	-	-	-	-	-	-	-	-	-	1.90	-	-	-	-	-
Hydrated chia*	-	-	-	-	-	-		-	-	-	7.62	-	-	3.81	1.90
Flaxseed	-	-	-	-	-	-	-	-	-	-	-	1.90	0.96	-	0.48
Water	52.38	52.38	52.38	52.38	52.38	52.38	52.38	52.38	52.38	52.38	46.66	52.38	52.38	49.53	50.96

Data are expressed as percentage (%) of total dough weight.

* The hydration process (chia flour: tap water ratio, 1:3) was carried out for 30 min at 20 °C.

Supplementary Table 2. Formulations of the experimental sourdoughs (DY = 210) made with rice and corn flours, and supplemented with quinoa and chestnut flours (10 or 20% of the flours total weight). Sourdoughs were fermented by *Weissella cibaria* P9 (initial cell density in dough of 7.0 log10 cfu/g) at 30 °C for 24 h.

	**s-CT**	**s-C10**	**s-C20**	**s-Q10**	**s-Q20**	**s-CQ10**
Rice	23.81	21.43	19.05	21.43	19.05	19.05
Corn	23.81	21.43	19.05	21.43	19.05	19.05
Chestnut	-	4.76	9.52	-	-	4.76
Quinoa	-	-	-	4.76	9.52	4.76
Water	52.38	52.38	52.38	52.38	52.38	52.38

Data are expressed as percentage (%) of total dough weight.
